# Austerity by Design: Comment on "Ensuring Global Health Equity in a Post-pandemic Economy"

**DOI:** 10.34172/ijhpm.2022.7754

**Published:** 2022-12-26

**Authors:** Indra de Soysa

**Affiliations:** Department of Sociology & Political Science (ISS), Norwegian University of Science & Technology (NTNU), Trondheim, Norway.

**Keywords:** Inequality, Population Health, Economic Development, Degrowth, Climate Change

## Abstract

Several scholars across many disciplines argue that neoliberal, free-market economic conditions drive inequalities, generating poverty and misery due to unfair austerity, ultimately affecting human health. Professor Labonté’s prescription is that we jettison these policies targeting economic growth and development for generating greater fairness for the world’s poor. This rejoinder argues contrarily that the criticism of neoliberal policies are misplaced, and that degrowth is really "self-imposed austerity," which will not benefit the poor. This rejoinder scrutinizes some simple stylized fact and assesses the soundness of the broader arguments. The evidence suggests clearly that becoming wealthy and following prudent economic policies is the best path to improving population health, equity, and other progressive outcomes. Badly required growth for the poor comes from free markets and good governance, and equity for the sake of fairness neither results in better health outcomes, nor an improved environment.

 Professor Labonté has written a thought-provoking editorial^1^ on the need to “reset” the global economy from its globalized, neo-liberal path to one where government intervention ensures greater fairness in terms of health equity and other favorable, progressive outcomes, not least the mitigation of climate change. Blaming globalization and neo-liberal policies for all the world’s ills is nothing new, nor just the preserve of public health scholars. From the climate crisis to pandemics, the underlying problem is blamed on the “unfairness” of neo-liberal policies, which generate inequalities (within and between societies), resulting in continued poverty and misery, authoritarianism, and environmental destruction. Many of the arguments tread a well-worn path, but they beg deeper scrutiny and supporting empirical evidence, particularly since Labonté’s main policy prescription for fighting neo-liberal austerity seems to be *degrowth, *which is a euphemism for “self-imposed austerity.” In an age of fake news, where populistic politicians everywhere offer simple solutions to complex problems, the need of the hour is well-considered theory and empirical evidence for guiding policy. This rejoinder, thus, will scrutinize some simple stylized fact and assess the soundness of the broader arguments, relying on the existing evidence in the specialized literature. There is much in Labonté’s article that is easy to agree with, and this author does not disagree with the larger claim that many global economic and policy processes are unfair to the poor, but what is questioned here is the empirical basis for relying on degrowth as a solution to questions of poverty, health, and fairness.

 First, I examine the issue of how the rich and poor have performed in the pre-pandemic world in terms of healthy life expectancy (HALE), which is perhaps the best way of evaluating population health because it assesses simultaneously how mortality and morbidity trends have evolved over the last three decades based on 369 known causes of mortality. According to the latest Global Burden of Disease study, all regions of the world have seen considerable improvements in HALE.^2^Figure 1 shows the regional trends in HALE (all cause) for both sexes above the age of 20.

**Figure 1:**
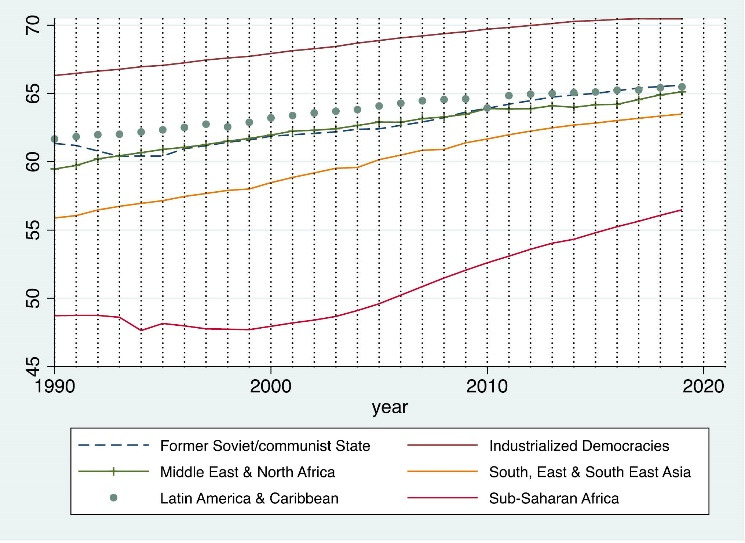
The Annual Average Regional Trends in Healthy Life Expectancy, 1990-2019.

 All regions have increased health-adjusted life years on the aggregate, and sub-Saharan Africa shows the steepest gains in the past two decades. Interestingly, after decades of so-called neo-liberal governance, Latin America and the Middle East and North Africa have similar HALE scores as the former Soviet States. There is little in this highly aggregated stylized view to suggest, thus, that egalitarian governance structures are a necessary condition for increasing healthy life and wellbeing. It is wealth created by good policy that allows both public and private investment in health to increase overall population health standings. While public action is important for achieving many health outcomes and human capital improvements, such as in Singapore, it is not at all clear that much could be achieved without growth of incomes and myriads private investment in health and education that happens when societies experience growth. Fortunately, countries such as China and Vietnam are following neoliberal policies, and their life improvements are palpable. Ultimately, levelling up the health gradient within poor countries is a noble objective, but increasing average wealth and health standards of the population at large is the surest path to achieving health equity. Neither higher government spending, nor equality alone, achieves better human health.^3,4^

 Consistent with the specialized literature, it is income levels, or wealth creation through economic growth, that matter for securing better life, not necessarily the distribution of wealth *per se*.^3,5,6^ There is by now a great deal of theoretical and empirical evidence suggesting that being open to global markets, where societies are governed by capitalist institutions and policies, increases the demand for public goods, such as health and education and drive better outcomes.^7,8^ At least one recent, careful empirical study shows quite unambiguously that higher amounts of foreign direct investment, and by extension openness to global capital, is strongly associated with higher HALE among the poorer countries, even after accounting for endogeneity.^9^ If indeed such global capitalist forces as foreign direct investment and trade associate strongly with country-level inequality, and inequality reduces average health, we would not expect to see such outcomes.^10^ In a footnote, Labonté acknowledges that growth can generate better outcomes for the poor, suggesting that the rich states should sacrifice their growth (degrowth) to allow higher growth for the poor. This is a rather surprising idea given that interdependence of economies is non-zero-sum. What the poor need are more markets and capital, which are linked intimately to growth among the rich. Today’s global slowdown, largely due to the slowdown of Chinese growth, is a sad but true reminder that degrowth will mean austerity by design.

 Labonté stresses “fairness,” putting his faith in government intervention for reducing inequality. Fairness, of course, is a rather slippery concept, especially if one tries to measure it. Economists usually use income or wealth inequality to measure fairness, differentiating inequalities of outcome, which occurs for many reasons, including natural causes (genes, for example) from inequalities of opportunity (institutional, structural factors). Figure 2 shows the trend in income inequality measured as the average Gini coefficient between the rich and poor worlds based on after tax disposable income.^11^

**Figure 2:**
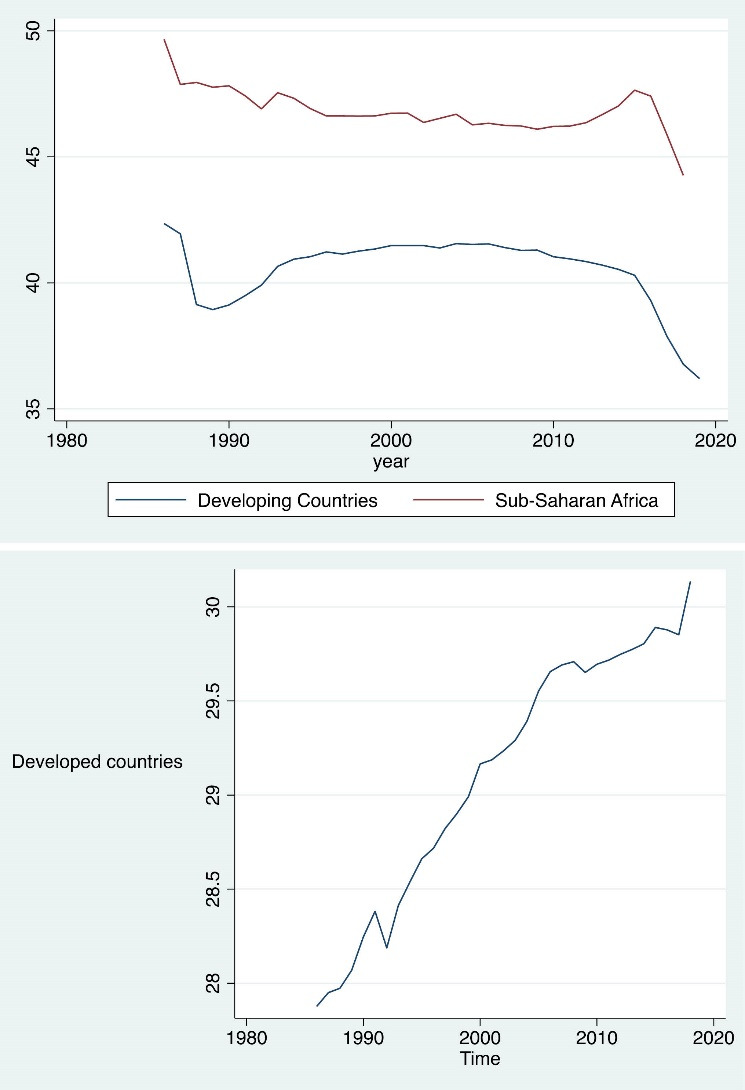
Regional Trends in the Gini Coefficient, 1985-2019.

 As seen there, the sub-Saharan Africa and developing countries as a group show higher inequality trends over the period of globalization compared with the industrialized countries as a group. Notice, however, that the developing countries show flatter, slightly downward trending inequality levels over this period, while the industrialized countries show a clear upward trend, albeit at a lower average Gini score. Clearly, the era of globalization has affected redistribution adversely mostly in the industrial world. However, this higher inequality is a by-product of high economic growth. The West is rich and has the financial and institutional wherewithal to reduce the worst harm generated by rising inequality—the poor do not. I am certain that most poor people living in places such as Cuba and North Korea would gladly give up their “equality” for less self-imposed austerity.

 Despite the upward trend in income inequality among the rich countries, it is precisely among them that the highest human and environmental health is found. Indeed, one widely recognized indicator used by global and local policy-makers, the Environmental Performance Index, suggests that being wealthy correlates best with local-level environmental outcomes, such as clean air and water and the protection of species. Clearly, the rich can afford to make the right adjustments.^12^ Again, it is the level of wealth produced by economic growth that seems to achieve the better outcomes for humans and the planet, not the pursuit of equality for the sake of levelling gradients. Several specialized studies on this subject show that greater openness to global markets and capitalistic economic policies produce far better environmental outcomes, particularly when it comes to reducing emissions and more efficient use of natural resources.^13^ Regardless, the world’s poor are literally dying to get to that region of the world where inequality is rising, but they care about the *absolute *improvements to their lives and the greater hope offered within wealthy societies rather than the *relative *deprivation they will inevitably have to face the minute they arrive there. What would be most fair to these desperate people, thus, is better economic conditions at home, given the absolute lack of appetite for more open borders, particularly in the more egalitarian societies in the West.

 Finally, I take a brief look at the data to assess if income equality and equal access to health (objective proxies of fairness) matter in terms of climate-harming emissions—climate change after all is the “mother of all problems.” If higher levels of wealth improve life conditions locally, does it endanger the global commons if one is less equal? Table presents results using an appropriate methodology for assessing the association between our variables of interest and environmental outcomes measured in terms of CO_2 _emissions per gross domestic product (GDP) and on a per capita basis.

**Table:** Fixed Effects Regressions of Egalitarian Governance and Greenhouse Gas Emissions, 1990-2019

**Dependent Variables**	**(1)**	**(2)**	**(3)**	**(4)**	**(5)**	**(6)**
	**CO**_2_ **/GDP**	**CO**_2_ **/GDP**	**CO**_2_ **/GDP**	**CO**_2_ **/pc**	**CO**_2_ **/pc**	**CO**_2_ **/pc**
Gini (disposable income) (ln)	-1.06***			-0.82***		
	(0.28)			(0.08)		
Equal access to education		0.08***			0.03***	
		(0.02)			(0.01)	
Equal access to health			0.03**			0.03***
			(0.02)			(0.01)
Income per capita (ln)	-0.49***	-0.41***	-0.41***	0.45***	0.35***	0.34***
	(0.07)	(0.07)	(0.07)	(0.04)	(0.02)	(0.02)
Urban population share (ln)	1.15***	1.11***	1.16***	0.42***	0.39***	0.40***
	(0.10)	(0.07)	(0.08)	(0.06)	(0.05)	(0.04)
Liberal democracy	0.04	0.29***	0.31***	0.13***	0.13***	0.13***
	(0.06)	(0.09)	(0.09)	(0.03)	(0.02)	(0.02)
Constant	3.72***	-0.93	-1.05	-1.42**	0.00	0.00
	(0.94)	(0.80)	(0.82)	(0.55)	(0.00)	(0.00)
Observations	3855	4789	4789	3812	4689	4689
Number of countries	169	171	171	168	170	170

Abbreviations: GDP, gross domestic product; pc, per capita. Standard errors in parentheses; X variables lagged 1 year; Year fixed effects estimated. *** *P* < .01, ** *P* < .05, * *P* < .1.

 The ordinary least squares estimates are based on Driscoll-Kraay standard errors robust to heteroskedasticity, first order serial correlation, and spatial dependence. As seen in columns 1-3, higher income inequality associates with lower emissions per GDP, and both equal access to education and health predict higher emissions per GDP. Consistent with arguments made by others, inequalities produce less greenhouse gases because governments that promote broad-based development (consumption) *ceteris paribus* necessarily generate higher emissions.^13,14^ The Gini’s negative effect is net of income level, which is also negative, suggesting that wealthier countries are environmentally efficient at producing wealth as are more unequal countries. In the next 3 columns (3-6), the inequality variables show the same effects when emissions are measured on a per capita basis, again supporting the view that societies that spread the wealth tend to generate higher emissions per head, presumably because of increased consumption. In this case, however, higher per capita incomes also produce more emissions per head, again, possibly because it captures higher consumption. The results taken across the table suggest that more egalitarian societies produce higher emissions. Arguments that suggest that “all good things go together” are too simplistic and wrongheaded, and ignoring difficult but necessary tradeoffs in policy-making should be addressed rationally rather than ideologically. Getting wealthy is good for health and wellbeing, but it may come at the cost of atmospheric pollution. Redistribution and equality of outcomes seem to unambiguously produce higher emissions possibly because of increased overall consumption—a good thing for human health, but perhaps a bad thing for planetary health. Yes, the rich should reduce consumption, and yes, the poor must catch up with increased growth, but as argued above, this is a complex problem unlikely to be solved by degrowth and by simply fixing “unfairness.”

 Again, it should be reiterated that there is much in Professor Labonté’s editorial that one can agree with, particularly the argument that the rich countries often take advantage of the poor. The poor clearly need more growth to improve life conditions. Such growth can only come from higher growth among the rich, which has pulled roughly a billion people out of abject poverty in the past decades, which is unprecedented. Indeed, organized interests among the rich often “fight the wrong enemy,” either due to perverse interests that seek to curtail capital outflows (outsourcing), or protect their own markets and jobs from foreign imports (agricultural trade barriers, tariffs, non-tariff barriers).^15^ Current calamities associated with rising autocrats are highly unlikely to be due to rising inequalities and dissatisfaction with neoliberal austerity since ordinary people are finding champions in snake-oil salesmen, such as Donald Trump and Vladimir Putin, whose only strategies seem to be to blame globalization and make empty promises about protecting domestic jobs and shelter from progressive social and environmental policies. Well-meaning people’s movements, whose banner Professor Labonté admirably carries, thus, should do well to avoid “austerity by design,” embracing growth-promoting economic freedoms and joining world markets—the West invented this wheel with great success already. The poor know this, but the rich seem to have forgotten.^16^

## Ethical issues

 Not applicable.

## Competing interests

 Author declares that he has no competing interests.

## Author’s contribution

 IdS is the single author of the paper.
